# Analysis of hip rotation ROM and strength in amateur soccer players: a cross-sectional study

**DOI:** 10.3389/fspor.2026.1837082

**Published:** 2026-06-01

**Authors:** Maria Figueroa-Mayordomo, Josep C. Benítez-Martínez, Joaquín Mas-Rivas, Javier González-Rosalén

**Affiliations:** 1 Faculty of Physiotherapy, Department of Physiotherapy, Universitat de Valencia, Valencia, Spain; 2 Faculty of Health Sciences, Department of Physiotherapy, Nutrition and Sports Sciences, Universidad Europea de Valencia, Valencia, Spain; 3 Head of Medical Staff, Sharjah Football Club, Sharjah, United Arab Emirates

**Keywords:** hand-held dynamometry, hip joint, hip rotator, range of motion, soccer, strength

## Abstract

**Introduction:**

This study aimed to examine variations in hip rotation range of motion (ROM) and strength when measured at 90° or 0° hip flexion in amateur soccer players, and to assess whether a standardized measurement protocol can be established. Additionally, it assessed the impact of normalizing strength values by body mass and lower leg length, and whether playing position affects hip rotator ROM and strength.

**Methods:**

A total of 56 amateur soccer players from the Valencian region were tested for maximal voluntary isometric contraction (MVIC) and ROM of hip rotator muscles, measured at both 0° and 90° hip flexion. ROM was measured using a digital inclinometer and strength was assessed using a handheld dynamometer. Statistical analyses included paired samples *t*-tests, effect size calculations, Pearson's correlation analysis, and one-way ANOVA to examine variations across playing positions.

**Results:**

Significant differences were found in hip internal rotation (IR) ROM at 90° vs. 0° flexion for both right and left hips (*p* = 0.001 and 0.011, respectively). However, the magnitude of these differences was limited, with higher ROM values at 90°, while external rotation (ER) differences were not significant. Strength measurements showed significant increases at 90° compared with 0° for IR and ER in both right and left hips (IR: *p* < 0.001; ER: *p* < 0.001 and 0.005, respectively). Normalized strength values also revealed significant differences for both variables across angles (*p* < 0.001–0.005). No significant differences were found between ROM and strength values when comparing playing positions.

**Conclusions:**

Findings support assessing strength in both positions, whereas ROM differences between positions were of limited magnitude and uncertain clinical relevance. These findings underscore the importance of context-specific assessment protocols. Normalizing strength values may not be necessary in this homogeneous population. No position-dependent differences in hip rotator ROM or strength were detected in this sample, suggesting that screening or normative values may not need to be position-specific for amateur male soccer players.

## 1 Introduction

Hip range of motion (ROM) and strength are critical components of functional movement, influencing athletic performance and injury prevention. The hip's ball-and-socket structure enables multiplanar movements, making its functional assessment integral to both clinical and research settings (1). Previous research has shown that hip biomechanics are influenced by the angle of hip flexion, as soft tissue and muscle activation patterns change dynamically with posture (1, 2). Specifically, hip internal rotation (IR) ROM is more restricted than external rotation (ER) when the hip is in an extended position, largely due to the increased tension in the surrounding soft tissue structures (3). These differences are particularly relevant in soccer, where kicking, rapid directional changes, and sprinting require effective hip to function across varying flexion angles.

In terms of strength, hip ER is primarily performed by the deep rotator muscle group (1, 3), whereas hip IR is produced by muscles acting as secondary internal rotators (1, 4, 5). Hip flexion angle also alters the moment arm of certain ERs, such as the superior portion of the gluteus maximus, with the moment arm changing from ER to IR function as hip flexion increases (6, 7). At 0° hip flexion, length-tension relationships differ from those at greater flexion angles, influencing force generation and joint stability (4). In this position, anterior capsuloligamentous structures, particularly the iliofemoral ligament, are taut, contributing to passive stability (8), whereas at 90° hip flexion, these ligaments become more lax, reducing passive stability but allowing for greater mobility. These changes affect both strength generation and ROM (9), with implications for soccer-specific tasks such as kicking and dribbling.

Alterations in hip rotator biomechanics have been linked to lower limb injuries in athletes. Soccer players with groin pain exhibited reduced hip ER ROM and altered biomechanics in symptomatic limbs, while strength remained unaffected (10). Additionally, deficits in hip ER strength have been associated with altered trunk, hip, and knee movement patterns, potentially increasing injury risk among athletes (11). These findings underscore the clinical importance of hip rotator function and possible influence of measurement protocols on interpreting biomechanical outcomes. Despite this, previous studies assessing hip rotator ROM (12–18) and strength (14, 17, 19) have used heterogeneous protocols, including variations in hip flexion angles, body position, stabilization, and normalization procedures. This methodological variability limits comparability across studies and hinders translation into clinical practice.

Although prior research has examined hip rotator ROM and strength at different hip flexion angles, limited evidence exists on how these factors interact within sport-specific populations or whether assessment protocols can be standardized for practical use. Amateur players represent the vast majority of soccer participants worldwide, with approximately 270 million individuals regularly playing the sport, making it the most popular sport in the world (20, 21), and are therefore likely to constitute a large proportion of individuals encountered in clinical settings. However, most biomechanical evidence is derived from elite or non-athletic cohorts (21). This is particularly relevant given that amateur soccer players exhibit higher overall injury incidence and recurrence rates than professional players (21, 22), indicating a greater clinical burden in this population. These differences raise concerns regarding the transferability of existing findings to amateur players. Compared to elite athletes, amateur players may demonstrate greater variability in training exposure, neuromuscular control, and physical conditioning, which may increase sensitivity to methodological factors such as testing position and stabilization (23). Consequently, the applicability of existing assessment protocols in this population remains unclear.

Previous studies comparing hip function across flexion angles have largely been conducted in controlled or homogeneous populations and therefore cannot determine whether previously reported differences reflect true biomechanical characteristics or methodological factors inherent to the testing protocol. In addition, prior studies have typically examined ROM or strength in isolation. Therefore, the present study simultaneously evaluates both outcomes under standardized conditions, including normalization procedures and consideration of playing position, within an amateur soccer population. This approach enables a more comprehensive assessment of whether commonly used protocols yield consistent and clinically meaningful results in a real-world setting. Accordingly, this study aims to examine variations in hip rotation ROM and strength at 90° or 0° hip flexion in amateur soccer players and to determine whether a standardized and clinically applicable assessment protocol can be established. Additionally, the study assessed the impact of normalizing strength values by body mass and lower leg length and whether playing position affects hip rotator ROM and strength. These findings may improve the reliability, efficiency, and practical applicability of hip assessments in amateur soccer players.

## 2 Materials and methods

A cross-sectional study was designed following the Strengthening and Reporting of Observational Studies in Epidemiology (STROBE) guideline (24). Ethics approval was obtained from the Universitat de Valencia (Spain) Ethics Committee (1662009173390_d2oa_1057_2173963). The data collection complied with the regulations of the current Spanish Data Protection Act “Organic Law 3/2018 regarding the Protection of Personal Data and guarantees of digital rights” (LOPDGDD).

### 2.1 Setting

This observational study was conducted in amateur soccer teams within the Valencian region. Before the observational study commenced, a measurement trial was done on a local amateur soccer club team. The aim of this trial was to obtain the necessary exposure to correct any discrepancies or limitations to the study design protocol before executing measurements on a bigger population.

The study was carried out during the 2022/23 and 2023/24 soccer seasons, with a duration from September to May, following a recruitment period of one month prior to the beginning of the seasons. The data collection occurred in designated testing areas within each institution's facilities. All assessments were conducted indoors on a stable treatment bench using the same testing equipment and protocols to ensure consistency across locations and minimize environmental variability. The research team consisted of 3 experienced clinicians in charge of different elements of the study. The main investigator was responsible for the study design as well as the participant intake, which consisted of collecting the informed consent forms from each participant and performing the patient history intake. A second investigator oversaw carrying out the strength and ROM measurements. A third investigator, blinded for measurements, was responsible for performing the statistical analysis.

### 2.2 Participants

A total of 56 healthy male amateur soccer players, aged 18–50, from the Valencian region participated in this study. For recruitment, a letter was sent out to various soccer clubs in the Valencian region stating the study's aim, methodology, and potential benefits. Players who agreed to participate were reported to the researchers and assessed for eligibility. All participants provided written informed consent before the initial evaluation.

The inclusion criteria were: (1) male amateur soccer players, (2) aged between 18 and 50, (3) players who were active participants in training sessions. The exclusion criteria were: (1) participants reporting current hip or groin pain, regardless of whether the pain resulted in time loss, (2) players with hip/groin injury in the preceding 4 months before study initiation (3) players with longstanding injuries (>6 weeks) in the lower extremities, (4) previous injury or surgery in the lumbopelvic or hip region in the past year, (5) players with >2/10 on the Visual Analogue Scale during ROM and strength testing protocols of this study.

Due to the lack of studies that evaluate the effect of hip flexion on hip ROM and strength, no available data was indicative of normative or minimal detectable change parameters to calculate the sample size needed for this study. Therefore, sample size was calculated using G*Power software (latest ver. 3.1.9.7; Heinrich-Heine-Universität Düsseldorf, Düsseldorf, Germany) (25). An *a priori* power analysis was used to estimate a clinically meaningful sample size. Said analysis was programmed for a statistical test of difference between two dependent means (matched pairs) with input parameters of: two-tailed, effect size of 0.5, Type I error probability (*α*) of 0.05, Type II error probability (1 − *β*) of 0.95, giving a sample size (n) of 54 participants.

### 2.3 Variables

Demographic and anthropometric values, including age (yrs.), weight (Kg), height (cm), lower leg length (m), and playing position, were logged in the data sheet by the data collector and are presented in the demographics table (see Table 1), represented in mean and standard deviation (SD). Body mass and height were measured using standard clinical procedures. Lower leg length was measured using the distance between the medial femoral condyle and medial malleolus of the participant.

**Table 1:** Participant characteristics (*n* = 56).

Variable	Mean (SD)	Range
Age (yrs)	25.50 (5.32)	19–46
Height (cm)	179.6 (6.7)	165–193
Weight (Kg)	77.5 (7.2)	64–90
Lower leg length (m)	0.368 (0.021)	0.320–0.420

SD, standard deviation.

The dependent variables for this study were ROM (degrees) and strength (Kg) values taken from the hip assessment. To normalize values between players, lower leg length and weight were taken into consideration for the strength values obtained (26, 27). The independent variables were hip flexion angle during measurements (0° and 90°) and participant playing position. The evaluator was a licensed physiotherapist with seven years of clinical experience in musculoskeletal assessment. Prior to data collection, the evaluator underwent extensive training in the use of both devices. Additionally, intra-rater relative reliability was assessed during the preliminary phase of the study using an intraclass correlation coefficient (ICC) analysis. The results indicated excellent reliability for both ROM and strength measurements of hip IR and ER across both testing positions (ICC > 0.90) (28). Exact ICC values are provided in Supplementary Tables S1 and S2. This preliminary trial also served to refine the assessment protocol, ensuring consistency and reliability in the measurements.

### 2.4 Data measurements

#### 2.4.1 Hip ROM

Hip ROM, specifically passive ROM (PROM), was measured using the Baseline® Digital Inclinometer (DI) (Fabrication Enterprises, White Plains, NY, USA). Studies have shown that the use of a DI provides reliable measurements of ROM (29–31), with an ICC ranging from 0.962 to 0.987 (30). A study by Santos 2012 observed that ROM measurements obtained with a DI were of higher reliability compared to those measured with a standard goniometer (29). The DI does not take specific anatomic structures into consideration when measuring ROM seeing as it measures surface inclination (in degrees) using gravity-sensitive sensors (29).

Testing was done on a training day before the training commenced. The inclinometer was calibrated against a protractor at 0° and 30° before each set of measurements, as per Mohammad et al. (31). ROM was assessed without a warm-up to avoid acute changes in passive mobility values prior to measurement. PROM was measured with the aid of the therapist assisting the movement to reach maximum PROM of the hip joint.

**Figure 1:**
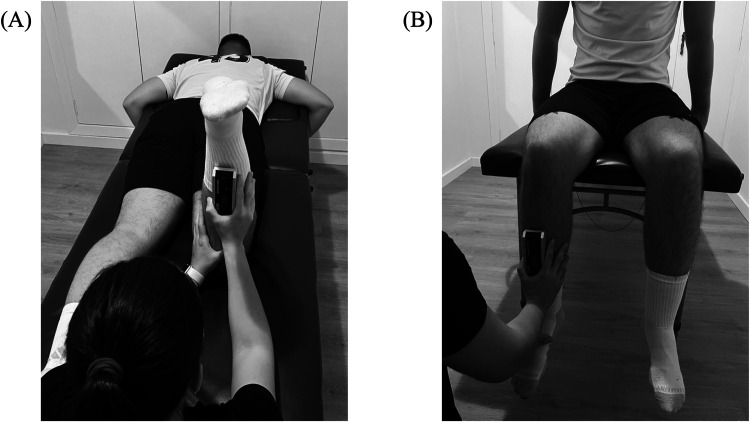
Patient positioning for range of motion testing. **(A)** Hip internal and external rotation with 0° hip flexion. **(B)** Hip internal and external rotation with 90° hip flexion.

Test orders were alternated for each participant to minimize order effects. Hip IR and ER was measured in prone (Figure 1A) and sitting (Figure 1B) position for both lower limbs. The measurement was repeated a total of three times in each direction, with 30 s rest between measurements. All three measurements were recorded on the Excel data collection sheet with the average value calculated for data analysis.

#### 2.4.2 Hip ROM at 90° hip flexion

Participants were seated at the edge of a treatment bench with the hip flexed to 90°. They were allowed to hold the sides of the bench to stabilize the pelvis and minimize compensatory movements. The DI was placed on the midportion of the tibia by the examiner. The examiner passively moved the limb to maximal hip IR and ER. Measurements were read by a second investigator and recorded.

#### 2.4.3 Hip ROM at 0° hip flexion

Participants were positioned prone on a treatment bench with the hip at 0° flexion and the knee flexed to 90°. A strap was placed around the lumbopelvic region to stabilize the pelvis and prevent compensatory movements. The DI was placed on the midportion of the tibia by the examiner. The examiner passively moved the limb to maximal hip IR and ER. Measurements were recorded.

#### 2.4.4 Hip strength

**Figure 2:**
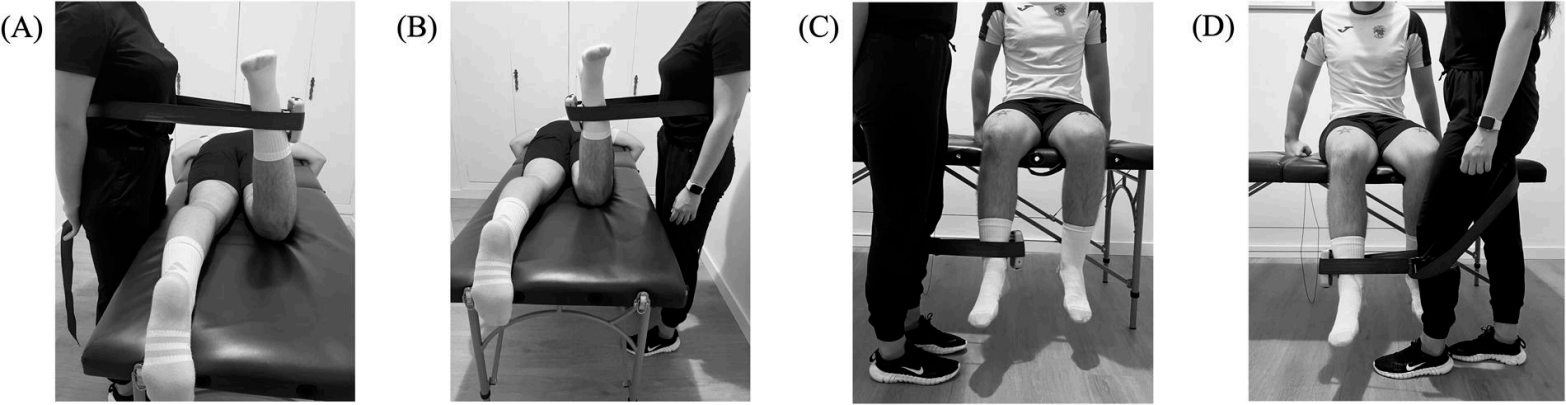
Patient positioning for maximal voluntary isometric contraction strength testing. **(A)** Hip internal rotation with 0° hip flexion. **(B)** Hip external rotation with 0° hip flexion. **(C)** Hip external rotation with 90° hip flexion. **(D)** Hip internal rotation with 90° hip flexion.

Isometric hip strength, specifically maximal voluntary isometric contraction (MVIC), was tested using a push hand-held dynamometer (HHD) (Lafayette Electronic Handheld Dynamometer, Model 01165), which measures force applied against the device (17, 32, 33). Previous studies have shown that externally stabilized dynamometry setups provide greater reliability when assessing high levels of isometric strength (32). Therefore, in the present study, the HHD was used in a strap-stabilized configuration to minimize examiner-related variability. By stabilizing the device and fixing it to the examiner's body, as opposed to the examiner holding the device, the influence of examiner strength and body size relative to the participant was reduced (32). Before testing, the HHD was calibrated using a certified 10 kg weight, following González-Rosalén et al. (32). Testing was performed with the participant lying on a treatment bench and the examiner in a standing position next to the bench. Measurements were taken for IR (Figure 2A) and ER (Figure 2B) at 0°, IR (Figure 2C) and ER (Figure 2D) at 90°.

Participants executed a warm-up before the testing commenced. The warm up consisted of active exercises, as stretching has been suggested to decrease exercise performance and doesn't significantly contribute to injury prevention (34). Participants performed 1 min of jumping jacks, 1 min of running on the spot and three submaximal isometric contractions in IR and ER directions with a resistance band. This, in turn, allowed them to familiarize with the correct execution of the tests (32). The participants were asked if the muscle contractions were pain-free (32). If participants experienced no pain during submaximal contractions, muscle testing proceeded; otherwise, they were excluded from the study.

The strength test began by testing hip IR and ER at 0° and 90° hip flexion. To avoid systematic bias and minimize potential order effects, test orders were alternated between participants. For testing of isometric strength participants were asked to perform MVIC for a duration of five seconds. This was done by instructing the participant to “apply as much force as possible throughout measurement … ready, push, push, push, push, relax”. This procedure was repeated a total of three times for each muscle group, with 60 s rest between measurements. All three measurements were recorded in the data sheet and the highest value was taken into consideration for the analysis of the data.

#### 2.4.5 Hip strength at 90° hip flexion

The participant was in a seated position on the edge of the treatment bench with 90° hip and knee flexion. For IR, the examiner was standing on the contralateral side of the testing limb. The HHD was placed three finger widths above the lateral malleolus, with the body strap around his lower limb at the height of his knee. The participant was then be asked to exert MVIC in IR. For ER, the examiner was standing on the ipsilateral side of the testing limb. The HHD was placed three finger widths above the medial malleolus, with the body strap as with IR. The participant was then be asked to exert MVIC in ER. The measurement was read and noted in the data collection sheet.

#### 2.4.6 Hip strength at 0° hip flexion

The IR strength at 0° hip flexion was measured with the participant in a prone lying position on the treatment bench. The examiner was standing on the contralateral side of the testing leg at approximately the level of the participant's knee. The testing leg was placed in 90° knee flexion. The HHD was positioned on the testing leg, three finger widths above the lateral malleolus and secured using a belt around the examiner's torso. The participant was then be asked to exert MVIC in IR, and the measurement was recorded.

The ER strength at 0° hip flexion was measured with the participant in the same prone position. The examiner stood on the ipsilateral side of the testing leg at approximately the level of the participant's knee. This positioning was selected to allow appropriate alignment of the dynamometer and direction of resistance relative to the rotational movement. The HHD was placed on the testing leg, three finger widths above the medial malleolus. The belt was placed around the examiner's torso. The participant was then be asked to exert MVIC in ER. The measurement was read and noted in the data collection sheet.

### 2.5 Statistical analysis

The variables assessed in this study were hip IR and ER peak MVIC and average ROM values, for both right and left lower limbs. Anthropometric data, ROM, and strength values were presented in mean ± SD. Normal distribution of the data was verified by the Kolmogorov–Smirnov test, with normality assumed obtaining a value of *p* > 0.05. The independent variables were hip flexion angle during measurements (0° and 90°) and participant playing position.

For within-subjects analysis, a paired samples *t*-test was administered to compare ROM and strength values obtained at 0° and 90° hip flexion, assuming a significance level of *p* < 0.05. For non-normally distributed data, the Wilcoxon signed-rank test was used. To control for multiple paired comparisons, Holm–Bonferroni adjusted *p*-values were calculated within related outcome families, and both raw and adjusted *p*-values are reported. Variables were normalized where appropriate. Confidence intervals were set at 95%.

Effect sizes were interpreted as follows: for Cohen's *d*, 0–0.19 (negligible), 0.20–0.49 (small), 0.50–0.79 (moderate), 0.80–1.19 (large), and ≥1.20 (very large); for effect size *r* from the Wilcoxon signed-rank test, 0.10–0.29 (small), 0.30–0.49 (medium), and ≥0.50 (large) (35). Measurement precision was further explored using minimal detectable change (MDC95) values derived from the reliability dataset. *post-hoc* power analyses were conducted for ROM comparisons based on observed effect sizes to assess the likelihood of Type II error.

To assess the correlation between measurements taken at 0° and 90° hip flexion, Pearson's correlation analysis was performed. Correlation coefficients (*r*) were interpreted as follows: 0.00–0.30 (negligible correlation), 0.30–0.50 (low), 0.50–0.70 (moderate), 0.70–0.90 (high), and 0.90–1.00 (very high) (36).

Finally, A one-way ANOVA was conducted to compare ROM and strength outcomes among defenders, midfielders, and attackers. Statistical significance was set at *p* < 0.05.

## 3 Results

Overall, 60 participants were initially considered for this study. However, four participants were deemed ineligible: two reported experiencing hip pain during the warm-up, and two did not attend the testing session. Consequently, a total of 56 participants were included in the study (mean age: 25.5 ± 5.3 years; height: 179.6 ± 6.7 cm; weight: 77.5 ± 7.2 kg). Participant characteristics are described in detail in Table 1.

Group values for ROM, strength and body mass and lower leg length-normalized strength values are reported in Table 2, presented in mean (SD). Additionally, ratio values obtained for hip IR to ER ROM and strength measurements at 90° and 0° hip flexion are reported in Table 2.

**Table 2:** Reference values for hip internal and external mean range of motion, strength, body mass and lower leg length-normalized strength values and internal/external rotation ratios.

	Range of Motion		Strength		Body mass and Lower Leg Length-Normalized Strength	
	Right Hip	Left Hip	Right Hip	Left Hip	Right Hip	Left Hip
	Mean (SD) degrees (°)	Mean (SD) degrees (°)	Mean (SD) (kg)	Mean (SD) (kg)	Mean (SD) (kg•m/kg)	Mean (SD) (kg•m/kg)
IR at 90°	24.97 (7.71)	26.43 (10.04)	26.47 (5.82)	24.92 (7.45)	.126 (.027)	.118 (.034)
IR at 0°	21.42 (9.49)	23.43 (9.92)	17.70 (4.50)	17.22 (4.27)	.084 (.022)	.082 (.020)
ER at 90°	32.53 (8.70)	31.00 (8.24)	22.61 (5.12)	21.11 (4.26)	.107 (.023)	.100 (.018)
ER at 0°	33.76 (8.58)	32.25 (7.50)	20.45 (4.68)	19.51 (4.41)	.097 (.021)	.093 (.020)
IR/ER ratio 90°	.791 (.231)	.888 (.335)	1.192 (.224)	1.186 (.296)	–	–
IR/ER ratio 0°	.653 (.269)	.729 (.274)	.887 (.214)	.905 (.219)	–	–

SD, standard deviation; IR, internal rotation; ER, external rotation; kg•m/kg: kg force × lower leg length/body mass: (kg)

Data are presented in mean ± SD. Values for strength are presented as maximal values, ROM values are presented as mean values.

Paired analysis of ROM measurements (Table 3a) revealed IR ROM was significantly greater at 90° for both the right (*p* = 0.004) and left hip (*p* = 0.033), with medium effect sizes (*r* = 0.41 and 0.34, respectively), and moderate correlation (*r* = 0.583 and 0.639, respectively). Estimated MDC95 values for hip IR ROM ranged from 2.27° to 3.70° across testing positions, with observed between-position mean differences of 3.00 to 3.55°. In contrast, ER ROM did not show significant differences between flexion angles. *post-hoc* power analysis for ER ROM comparisons indicated low statistical power (1 − *β* right: 0.26; 1 − *β* left: 0.24).

**Table 3a:** Comparison of group mean values for average hip external and internal rotation range of motion values comparing testing positions at 90° and 0° hip flexion.

Comparison	Range of Motion						
	Difference between Mean Values (95% CI)	Standard Error	*p*	Adjusted *p*	*r*/Cohen's *d*	Pearson's Correlation Coefficient (*r*)	Correlation *p*-value
IR R 90°–IR R 0°	3.55 (1.41, 5.70)	1.070	**.** **001** * ^a^	**.** **004** *	0.41	0.583	**<0** **.** **001** **
IR L 90°–IR L 0°	3.00 (.73, 5.27)	1.133	**.** **011** * ^a^	**.** **033** *	0.34	0.639	**<0** **.** **001** **
ER R 90°–ER R 0°	−1.23 (−3.03,.57)	0.898	.175^b^	.350	−0.18	0.697	**<0** **.** **001** **
ER L 90°–ER L 0°	−1.25 (−3.27,.78)	1.010	.223^b^	.223	−0.17	0.541	**<0** **.** **001** **
IR/ER R 90°–IR/ER R 0° ratio	.138 (.076,.202)	0.032	**<** **.** **001** ** ^b^	**<** **.** **001** **	0.59	0.560	**<0** **.** **001** **
IR/ER L 90°–IR/ER L 0° ratio	.159 (.076,.243)	0.042	**<** **.** **001** ** ^b^	**.** **002** *	0.51	0.479	**<0** **.** **001** **

CI, confidence interval; IR, internal rotation; ER, external rotation; R, right hip; L, left hip. Adjusted *p*-values were calculated using the Holm–Bonferroni method.

a Wilcoxon signed-rank test; effect size reported as *r*.

b Paired samples *t*-test; effect size reported as Cohen's *d*.

* *p* < .05.

** *p* < .001.

Significant differences were observed across all strength variables. Hip IR strength was significantly greater at 90° compared to 0° hip flexion (*p* < 0.001; right: *d* = 1.90, left: *d* = 1.14), with low to moderate correlations (right: *r* = 0.626, left: *r* = 0.442). Similarly, ER strength was higher at 90° than at 0° for both the right (*p* < 0.001; *d* = 0.54) and left hip (*p* = 0.005; d = 0.40), with moderate correlations (right: *r* = 0.669, left: *r* = 0.562). Hip IR and ER strength are both significantly higher at 90° hip flexion, highlighting distinct functional demands at different angles (see Table 3b).

**Table 3b:** Comparison of group mean values for average hip internal and external rotation maximal voluntary isometric contraction strength values comparing testing positions at 90° and 0° hip flexion.

Comparison	Strength						
	Difference between Mean Values (95% CI)	Standard Error	*p*	Adjusted *p*	Cohen's *d*	Pearson's Correlation Coefficient (*r*)	Correlation *p*-value
IR R 90°–IR R 0°	8.77 (7.53, 10.06)	0.618	**<** **.** **001** ** ^a^	**<** **.** **001** **	1.90	0.626	**<0.001** **
IR L 90°–IR L 0°	7.70 (5.89, 9.50)	0.902	**<** **.** **001** ** ^a^	**<** **.** **001** **	1.14	0.442	**0** **.** **002** *
ER R 90°–ER R 0°	2.17 (1.09, 3.24)	0.535	**<** **.** **001** ** ^a^	**<** **.** **001** **	0.54	0.669	**<0** **.** **001** **
ER L 90°–ER L 0°	1.60 (.51, 2.69)	0.543	**.** **005** * ^a^	**.** **005** *	0.40	0.562	**<0** **.** **001** **
IR/ER R 90°–IR/ER R 0° ratio	.305 (.241,.370)	0.033	**<** **.** **001** ** ^a^	**<** **.** **001** **	1.26	0.377	**0** **.** **004** *
IR/ER L 90°–IR/ER L 0° ratio	.281 (.192,.370)	0.190	**<** **.** **001** ** ^a^	**<** **.** **001** **	0.83	0.162	0.229

CI, confidence interval; IR, internal rotation; ER, external rotation; R, right hip; L, left hip. Adjusted *p*-values were calculated using the Holm–Bonferroni method.

a Paired Samples *t*-test; effect size reported as Cohen's *d*.

* *p* < .05.

** *p* < .001.

ROM ratios demonstrated medium effect sizes (*d* = 0.59 and 0.50) and moderate correlations (*r* = 0.560 and 0.479). In contrast, strength ratios exhibited very large to large effect sizes (*d* = 1.26 and 0.83) and low to negligible correlations (*d* = 0.377 and 0.162). ROM and strength ratios are significantly higher (*p* < 0.001) at 90° hip flexion.

Normalization of strength values for body mass and lower leg length revealed a significant increase in all measurements at 90° hip flexion (*p* < 0.001–0.005). IR strength demonstrated very large effect sizes (*d* = 1.98 and 1.40) with low to moderate correlations (*r* = 0.650 and 0.409). ER strength exhibited small to medium effect sizes (*d* = 0.55 and 0.39) with low to moderate correlations (*r* = 0.635 and 0.472) (Table 3c). Normalization by body mass and lower leg length does not alter the trend of higher strength values at 90°, reinforcing functional differences between testing positions.

**Table 3c:** Comparison of group mean values for average hip internal and external body mass and lower leg length-normalized strength values comparing testing positions at 90° and 0° hip flexion.

Comparison	Body Mass and Lower Leg Length-Normalized Strength						
	Difference between Mean Values (95% CI)	Standard Error	*p*	Adjusted *p*	Cohen's *d*	Pearson's Correlation Coefficient (*r*)	Correlation *p*-value
IR R 90°–IR R 0°	.041 (.036,.047)	0.003	**<** **.** **001** ** ^a^	**<** **.** **001** **	1.98	0.650	**<0** **.** **001** **
IR L 90°–IR L 0°	.036 (.028,.044)	0.004	**<** **.** **001** ** ^a^	**<** **.** **001** **	1.44	0.409	**<0** **.** **001** **
ER R 90°–ER R 0°	.010 (.005,.015)	0.003	**<** **.** **001** ** ^a^	**<** **.** **001** **	0.55	0.635	**<0** **.** **001** **
ER L 90°–ER L 0°	.019 (.002,.013)	0.003	**.** **005** * ^a^	**.** **005** *	0.39	0.472	**<0** **.** **001** **

CI, confidence interval; IR, internal rotation; ER, external rotation; R, right hip; L, left hip. Adjusted *p*-values were calculated using the Holm–Bonferroni method.

a Paired samples *t*-test; effect size reported as Cohen's *d*.

* *p* < .05.

** *p* < .001.

One-way ANOVA did not reveal significant differences in ROM (*p* = 0.06–0.46) or strength (*p* = 0.14–0.76) values across different playing positions (defenders: *n* = 19, midfielders: *n* = 16, and attackers: *n* = 17). Specific values for IR and ER at both testing positions across playing positions are provided in Supplementary Tables S3 and S4.

## 4 Discussion

The primary aim of this study was to examine variations in hip rotation ROM and strength when measured at 90° or 0° hip flexion in amateur soccer players and to assess whether a standardized measurement protocol can be established. Additionally, the study aimed to assess the impact of normalizing strength values by body mass and lower leg length, as well as whether playing positions affect hip rotator ROM and strength. The key findings indicate that hip IR ROM is significantly greater at 90° hip flexion, whereas ER ROM remains unchanged between positions. Strength measurements for both IR and ER were also higher at 90°, indicating that flexion angle influences hip rotator function. Additionally, ROM and strength ratios were significantly greater at 90°, with strength ratios showing larger effect sizes. Correlations across positions were low to negligible. Normalization by body mass and lower leg length did not modify these trends, suggesting that positional differences persist regardless of scaling. Lastly, the one-way ANOVA indicated no significant differences in ROM or strength measurements among players in different playing positions. These findings suggest that hip rotator function may not differ substantially by playing position within this sample.

### 4.1 Range of motion

This study is the first, to the authors' knowledge, to simultaneously compare hip rotator ROM and strength at different hip flexion angles in amateur soccer players. The results indicate that hip IR ROM is significantly greater when measured at 90° compared to 0° hip flexion. In contrast, ER ROM measurements at 90° and 0° hip flexion did not reach statistical significance.

Although no significant differences were observed in ER ROM between positions, *post-hoc* power analysis indicated low statistical power (1 − *β* = 0.24–0.26). Therefore, these findings should be interpreted with caution, as the study may have been underpowered to detect small effects and the possibility of a Type II error cannot be excluded. Despite the significant differences in IR ROM, it is important to note that IR values exhibited substantial interindividual variability, as reflected by a high standard deviation, leading to a non-normally distributed variable. Moreover, the observed between-position differences in IR ROM (right: 3.55°; left: 3.00°) were within the range of the estimated MDC95 values (2.27°–3.70°), indicating that these differences are close to the threshold of measurement error and should be interpreted with caution. The moderate correlations observed suggest that, while positional differences exist, their practical relevance for individual assessments remains unclear and warrants further investigation.

Comparisons with existing literature highlight inconsistencies in ROM behavior across different testing positions. A previous study assessing hip rotation ROM in sitting and prone positions for both right and left hips reported significantly greater IR and ER ROM in the prone position (38). These findings contrast with the present study, where IR ROM was greater at 90° of hip flexion, possibly due to differences in participant characteristics, testing procedures (including stabilization and positioning), and the effects of hip flexion angle on capsuloligamentous tension and muscle length-tension relationships. Similarly, Simoneau et al. found significant differences in ER ROM between testing positions, whereas IR ROM remained unchanged (8). This is in direct contrast to our findings, where IR ROM demonstrated significant differences while ER ROM did not. The discrepancies could be attributed to intersubject variability in hip rotational mobility across different postures, as well as differences in sample characteristics and methodological approaches. These findings support further research to determine whether specific hip flexion angles should be prioritized in clinical and athletic assessments.

### 4.2 Strength

#### 4.2.1 Internal rotation

Results of this study indicate that, for hip IR, strength significantly increases when measured at 90° hip flexion compared to 0° for both right and left hips, with large effect sizes seen in the statistical analysis. In addition to these large effect sizes, moderate to low correlations were found, reinforcing the notion that functional differences exist between the two testing positions. This could be explained by the anatomical origins of the hip IRs, as it is challenging to define a primary IR in the horizontal plane (1, 4). Muscle modeling studies done on cadaveric specimens suggest that secondary IRs, particularly the anterior fibers of the gluteus maximus, contribute substantially to IR function, with an approximately eight-fold increase (4) in IR leverage at 90° of hip flexion compared to 0° (4, 6, 9). This shift occurs as the gluteus maximus transitions from functioning as an ER to an IR beyond a 50° hip flexion (6).

Although previous studies have compared IR and ER strength values, only four have examined these values at different testing positions (17, 38–40). These studies, consistent with our findings, demonstrate that IR values significantly increase when measured at 90° hip flexion compared to 0°. Notably, Johnson (38) and Lindsay (40) assessed strength torque values, while Bloom (39) and Hoglund (17) measured maximum isometric strength in healthy male and female participants (17, 38–40). Subjective feedback from participants in the present study aligns with findings from one of these studies, indicating that generating force was more challenging at 0° hip flexion (39). However, given the lack of research specifically focusing on athletic populations, further investigation is necessary to determine the implications of these differences in sports performance and injury prevention.

#### 4.2.2 External rotation

As for ER, higher strength values were observed at 90° compared to 0° hip flexion. Furthermore, the moderate correlation observed between ER strength values at these two positions suggests that, similar to IR strength, measurement angle influences functional output, with each position providing distinct information. Previous studies focusing on hip kinesiology identified the gluteus maximus as the strongest ER muscle of the hip, with the primary ER muscles generally positioned posterolateral to the hip joint's vertical axis of rotation in the anatomical position (4, 9). These studies suggest that as hip flexion increases, the moment arms of the ER muscles decrease, leading to a potential reduction in ER strength (4, 6, 9). Specifically, the anterior fibers of the gluteus maximus and tensor fascia lata are more actively recruited as IRs and minimally engaged as ERs beyond approximately 50° of hip flexion (6). Additionally, prior research indicates that the ER muscle group is fully engaged during unipodal cutting motions, with the hip closer to 0° flexion, which are common in sports requiring rapid changes of direction such as soccer (4).

However, the present findings do not entirely align with these theoretical models, as ER strength was greater at 90° hip flexion. The literature presents mixed findings regarding ER strength across hip positions. Johnson (38) and Bloom (39) found no significant difference in hip ER strength values between different testing positions (38, 39), whereas Lindsay (40) and Hoglund (17) reported higher ER strength values at 90° compared to 0° hip flexion, consistent with the results of this study (17, 40).

These discrepancies across studies may be attributed to differences in participant characteristics, including sex, age, and athletic background, as well as methodological variations, such as the use of supine vs. prone testing positions for hip rotation assessments at 0° hip flexion. Furthermore, testing in a seated position may provide greater trunk and pelvic stabilization, facilitating more effective force transmission and potentially higher strength values despite less favorable moment-arm mechanics. Further research is needed to clarify these inconsistencies and explore their clinical and performance-related implications.

### 4.3 Ratios

Ratios are commonly used as indicators of injury risk. The findings of this study demonstrate that both ROM and strength ratios were significantly higher when measured at 90° hip flexion compared to 0°. Additionally, ROM ratios exhibited low to moderate correlations with each other, while strength ratios across these positions showed low to negligible correlations. These findings reinforce the notion that different measurement positions yield distinct ratio values.

The present results align with those reported by Lindsay (40), who suggest that differences in IR/ER ratios may be attributed to the aforementioned changing moment arm of specific hip rotators, such as the piriformis muscle, which alters its contribution to rotation as hip flexion increases (38). Additionally, Kim (41) identified a significant difference in hip IR/ER ROM and strength ratios between participants with muscle imbalance and those with normal values, indicating a decrease in dynamic balance ability in those with muscle imbalances (41).

Furthermore, studies investigating injured populations have highlighted the impact of injury on ROM. Nevin (13) reported that Gaelic football players with injuries exhibited significantly reduced hip IR and ER ROM values compared to their uninjured counterparts (*p* < 0.05) (13). Similarly, Tak (42) found that both elite and amateur soccer players showed similar hip IR and ER ROM values, whereas those in the injured group demonstrated a significant reduction in overall ROM (*p* < 0.05) (42). However, it is important to note that ROM values in these studies were assessed using different testing protocols, with Nevin (13) measuring ROM in a side-lying position with the hip in extension, while Tak (42) measured IR in the prone position and ER in the supine position. These methodological differences are particularly relevant in light of the present findings, as it remains uncertain whether variations in hip flexion angle meaningfully influence ROM values. Therefore, discrepancies between studies and between injured and uninjured populations may be partially influenced by testing position, rather than solely reflecting true biological differences. This highlights the importance of standardized measurement procedures when interpreting and comparing ROM outcomes across studies.

The observed variations in ROM and strength ratios with injury presence underscore the potential value of further investigation to refine injury prevention and rehabilitation strategies. A more functional approach to hip rotation assessments may help inform targeted interventions aimed at reducing injury risk and optimizing athletic performance.

### 4.4 Limitations

This study has several limitations that should be acknowledged. First, the study was limited to male participants, which restricts the generalizability of the findings to other populations, such as female athletes. The potential benefits of normalizing strength values may differ in female participants or heterogeneous populations, which could not be evaluated in this study due to the teams available to the researchers. Additionally, the study focused exclusively on amateur soccer players, without including participants from different levels of play. This narrow scope limits the applicability of the results across different types of players and competitive levels. Furthermore, the assessments were conducted at each institution's facilities, which may have introduced variability in testing conditions. This limitation was addressed by the investigators through the thorough protocolization of assessment procedure. However, such variability could affect the consistency of the measurements and the comparability of results across different locations. In addition, pelvic stabilization differed between testing positions, with strap stabilization used in the prone position and self-stabilization in the seated position. This may have allowed varying degrees of pelvic motion and represents a potential confounding factor when comparing ROM between positions. Future research should address these limitations by including a more diverse participant pool and standardizing testing environments.

### 4.5 Future investigations

Future research should aim to address the limitations of this study by including a more diverse participant pool, encompassing both male and female athletes, differing age groups, as well as professional and amateur players across various playing positions and sports.

Normalizing strength values by body mass and lower leg length appears to be unnecessary in this population, as our results indicate that outcomes are similar to those obtained using raw values. However, the literature lacks studies that analyze these normalized values across different population types. Future research focusing on strength value differences among various populations could provide a broader reference framework and help determine whether normalization is necessary for heterogeneous participant groups.

Moreover, no position-dependent differences were observed. However, these findings should not be extrapolated to broader training program design.

## 5 Conclusions

This study investigated differences in hip rotation ROM and strength at 90° and 0° hip flexion, as well as the influence of normalization procedures and playing position in amateur soccer players. Hip ROM was minimally affected by testing position, with differences of limited magnitude that were close to the threshold of measurement error, indicating that the added value of assessing ROM in both positions remains uncertain. However, strength outcomes differed significantly between positions, suggesting that testing at both 0° and 90° hip flexion captures distinct aspects of hip rotator function and should be considered for comprehensive assessment. Normalization of strength values to body mass and lower leg length did not improve the interpretability of results, indicating that raw values may be sufficient in this population. Additionally, no clinically meaningful differences were observed across playing positions. These findings support the inclusion of multiple testing positions for strength assessment, with implications for the standardization of hip evaluation protocols in healthy amateur soccer players.

## Data Availability

The raw data supporting the conclusions of this article will be made available by the authors, without undue reservation.
