# Supplementary material for: Analysis of hip rotation ROM and strength in amateur soccer players: a cross-sectional study
Source: Front Sports Act Living. 2026 Jun 1;8:1837082. doi: 10.3389/fspor.2026.1837082 (PMC13267181; doi:10.3389/fspor.2026.1837082)
Supplement: Supplementary file 1 [file Table1.docx]

Supplementary Material

**Supplementary Table 1**. Intra-Rater Intraclass correlation coefficients (ICC) for repeated measures of hip internal (IR) and external (ER) rotation average range of motion at different hip flexion positions in 15 participants.

| **ROM** | | |
| --- | --- | --- |
|  | Right Hip (ICC (95% CI)) | Left Hip (ICC (95% CI)) |
| IR at 90˚ | .982 (.958-.994) | .972 (.934-.990) |
| IR at 0˚ | .950 (.882-.982) | .976 (.944-.991) |
| ER at 90˚ | .977 (.944-.991) | .974 (.938-.991) |
| ER at 0˚ | .967 (.921-.988) | .976 (.943-.991) |
| ICC: intraclass correlation coefficient; CI: confidence interval; IR: internal rotation; ER: external rotation; 90˚: 90˚ hip flexion; 0˚: 0˚ hip flexion. | | |

**Supplementary Table 2.** Intra-Rater Intraclass correlation coefficients (ICC) for repeated measures of hip internal (IR) and external (ER) rotation maximum isometric strength in 15 participants.

| **Strength** | | |
| --- | --- | --- |
|  | Right Hip (ICC (95% CI)) | Left Hip (ICC (95% CI)) |
| IR at 90˚ | .939 (.856-.978) | .927 (.827-.974) |
| IR at 0˚ | .903 (.771-.965) | .951 (.883-.982) |
| ER at 90˚ | .902 (.767-.964) | .950 (.881-.982) |
| ER at 0˚ | .935 (.847-.977) | .914 (.795-.969) |
| ICC: intraclass correlation coefficient; CI: confidence interval; IR: internal rotation; ER: external rotation; 90˚: 90˚ hip flexion; 0˚: 0˚ hip flexion. | | |

**Supplementary Table 3.** One-way Analyses of Variance on average internal and external hip rotation ROM in terms of field playing position (defender, midfielder, or attacker).

|  | | Defender  (n =19) | | Midfielder  (n = 16) | | Attacker  (n = 17) | | F (2,49) | *p* |  |
| --- | --- | --- | --- | --- | --- | --- | --- | --- | --- | --- |
|  | | M | SD | M | SD | M | SD |  |  |  |
| ROM R IR 90˚ | | 26.26 | 7.86 | 23.29 | 7.85 | 26.50 | 7.70 | 0.869 | 0.43 |  |
| ROM L IR 90˚ | | 27.83 | 11.48 | 24.00 | 7.72 | 27.92 | 11.01 | 0.781 | 0.46 |  |
| ROM R ER 90˚ | | 33.66 | 9.63 | 29.94 | 6.31 | 34.16 | 10.10 | 1.104 | 0.34 |  |
| ROM L ER 90˚ | | 32.82 | 7.98 | 26.94 | 5.81 | 33.10 | 9.83 | 3.069 | 0.06 |  |
| ROM R IR 0˚ | | 23.22 | 10.62 | 19.22 | 8.79 | 24.18 | 7.73 | 1.348 | 0.27 |  |
| ROM L IR 0˚ | | 23.81 | 11.96 | 20.26 | 8.63 | 27.67 | 7.92 | 2.362 | 0.11 |  |
| ROM R ER 0˚ | | 34.40 | 10.72 | 30.75 | 7.20 | 35.56 | 7.26 | 1.377 | 0.26 |  |
| ROM L ER 0˚ | | 33.86 | 8.65 | 30.01 | 6.35 | 33.26 | 7.26 | 1.263 | 0.29 |  |
|  | M: mean (presented in degrees); SD: standard deviation; F(df between groups, df within groups); be ROM: range of motion; R: right hip; L: left hip; IR: internal rotation; ER: external rotation.  *p* < 0.05*, *p* < 0.001**** | | | | | | | | | |

**Supplementary Table 4.** One-way Analyses of Variance on maximum internal and external hip rotation strength in terms of field playing position (defender, midfielder, or attacker).

|  | | Defender  (n =19) | | Midfielder  (n = 16) | | Attacker  (n = 17) | | F (2,49) | *p* |  |
| --- | --- | --- | --- | --- | --- | --- | --- | --- | --- | --- |
|  | | M | SD | M | SD | M | SD |  |  |  |
| Strength R IR 90˚ | | 24.68 | 5.32 | 26.49 | 4.74 | 27.08 | 7.01 | 0.849 | 0.43 |  |
| Strength L IR 90˚ | | 22.28 | 6.98 | 27.19 | 9.24 | 24.08 | 5.01 | 2.026 | 0.14 |  |
| Strength R ER 90˚ | | 21.06 | 5.07 | 23.98 | 4.55 | 21.83 | 4.25 | 1.795 | 0.18 |  |
| Strength L ER 90˚ | | 19.62 | 3.75 | 21.82 | 5.49 | 20.82 | 2.43 | 1.296 | 0.28 |  |
| Strength R IR 0˚ | | 16.90 | 3.94 | 17.53 | 3.33 | 18.59 | 5.83 | 0.641 | 0.53 |  |
| Strength L IR 0˚ | | 16.51 | 3.86 | 18.21 | 3.98 | 17.41 | 5.19 | 0.657 | 0.52 |  |
| Strength R ER 0˚ | | 18.68 | 4.39 | 20.56 | 4.47 | 21.58 | 4.98 | 1.835 | 0.17 |  |
| Strength L ER 0˚ | | 18.62 | 3.93 | 19.68 | 4.51 | 19.44 | 5.02 | 0.275 | 0.76 |  |
|  | M: mean (presented in Kg); SD: standard deviation; F(df between groups, df within groups); R: right hip; L: left hip; IR: internal rotation; ER: external rotation.  *p* < 0.05*, *p* < 0.001**** | | | | | | | | | |
